# Leptin independently predicts development of sepsis and its outcome

**DOI:** 10.1186/s12950-017-0167-2

**Published:** 2017-09-11

**Authors:** Sofie Jacobsson, Peter Larsson, Göran Johansson, Margareta Norberg, Göran Wadell, Göran Hallmans, Ola Winsö, Stefan Söderberg

**Affiliations:** 1 Department of Surgical and Perioperative Sciences, Anesthesiology and Intensive Care Medicine, Umeå University Hospital, Umeå University, S-901 85 Umeå, Sweden; 2 Department of Public Health and Clinical Medicine, Epidemiology and Global Health, Umeå University, Umeå, Sweden; 3 Department of Clinical Microbiology, Umeå University, Umeå, Sweden; 4 Department of Public Health and Clinical Medicine, Nutritional Research, Umeå University, Umeå, Sweden; 5 Department of Public Health and Clinical Medicine, Medicine, Umeå University, Umeå, Sweden

**Keywords:** Sepsis, Leptin, Adiponectin, Obesity, Case-referent study, Sex

## Abstract

**Background:**

Sepsis is a life-threatening condition and obesity is related to the clinical outcome. The underlying reasons are incompletely understood, but the adipocyte derived hormones leptin and adiponectin may be involved.

**Methods:**

Patients aged 18 years or more with documented first time sepsis events were included in a nested case-referent study if they had participated in previous health surveys. Two matched referents free of known sepsis were identified. Circulating levels of leptin and adiponectin were determined in stored plasma, and their impact on a future sepsis event and its outcome was evaluated.

**Results:**

We identified 152 patients (62% women) with a sepsis event and a previous participation in a health survey. Eighty-three % had also blood samples from the acute event. Hyperleptinemia at health survey associated with a future sepsis event (OR 1.77, 95% CI 1.04–3.00) and with hospital death. After adjustment for BMI leptin remained associated with sepsis in men, but not in women. High levels in the acute phase associated with increased risk for in hospital death in women (OR 4.18, 95% CI 1.17–15.00), while being protective in men (OR 0.05, 95% CI 0.01–0.48). Furthermore, leptin increased more from baseline to the acute phase in men than in women. Adiponectin did not predict sepsis and did not relate to outcome.

**Conclusions:**

Hyperleptinemia independently predicted the development of sepsis and an unfavourable outcome in men, and inertia in the acute response related to worse outcome.

## Background

Sepsis is a life threatening condition with an increasing incidence globally [1]. In parallel, the prevalence of obesity is increasing, and obesity is common among sepsis patients and has been associated with both worse and favourable outcomes [2–4]. The underlying reasons are incompletely understood, but the adipose tissue may be an important modulator of inflammation and immunity via production of vasoactive substances such as cytokines and other peptides collectively known as adipokines [5, 6].

The adipokines leptin and adiponectin are involved in the inflammatory process, and they may modulate key processes during sepsis development such as cytokine production, immune cell proliferation and endothelial function through interaction with other cytokines and their receptors [7–10].

However, there are conflicting results regarding these adipokines and the outcome from sepsis [11–21]. Although adipokines have been associated with the prognosis of sepsis, it is not clear if adipokines promotes an infectious process to become septic or are related to the outcome of the septic event [22].

In this study, we aimed to assess the risk for sepsis and its outcome related to circulating levels of adipokines both at baseline and at the acute phase. Furthermore, if these associations differed between sexes.

## Methods

Between 1 March 1988 and 31 October 2008, a total of 797 patients were admitted with a diagnosis of sepsis at the Intensive Care Unit, Umeå University Hospital, Sweden. The diagnosis of sepsis and the severity of sepsis were confirmed retrospectively by reviewing hospital records, including results from biochemical, microbiological, and radiological examinations.

Prior to the septic event, 152 of the 797 had participated in one of four population-based health studies in Northern Sweden: the Västerbotten Intervention Program (VIP), the Northern Sweden MONItoring Of trends and Determinants in CArdivascular Disease (MONICA) survey, the Mammary Screening Program (MSP), and the Northern Sweden Maternity Cohort (NSMC). The contribution of cases from each survey was 81 (VIP), 4 (MONICA), 43 (MSP), and 24 (NSMC).

VIP is an ongoing community intervention program targeting cardiovascular disease and diabetes prevention [23]. Subjects are asked to participate in a health survey at their primary health centre at the ages of 30, 40, 50 and 60 years. However, those aged 30 are no longer invited because of a lack of resources. The participation rate was initially 55% but has increased and is now approximately 65%. The total number of unique individuals surveyed in VIP was 85,600 as of 31 December 2009.

MONICA consists of randomly selected individuals aged 25–74 years from the counties of Norr- and Västerbotten, who were invited to participate in a health study. The study has been repeated six times until 2009 and 10,300 unique persons participated (74% of those invited) [24].

Data for the MSP cohort, consisting of 28,700 women, were collected between 1995 and 2006 when the women attended their regular mammography exam and were asked to donate blood samples for research. In addition, anthropometric measurements were taken.

In VIP, MONICA and MSP, participants were asked to donate blood to the Northern Sweden Biobank for future research, and blood was stored at −80 °C until further analysis. Participants were fasting before sampling for a minimum of 4 h (extended to 8 h in 1992).

The Northern Sweden Maternity Cohort (NSMC) consists of all women in the study area who were screened for rubella immunity during pregnancy, and the total number of participants were 91,000 as of 31 December 2009. Remaining samples from rubella screening as well as from routine clinical serological and viral analyses have been stored at −20 °C since 1975 at the Department of Virology, Umeå University.

For each septic case, two referents from the same cohort without any episode of sepsis and being alive at the date of the case admission to ICU were chosen and matched for age (±2 years), gender, and time of blood sampling (±30 days). Matching on smoking (y/n) was incomplete due to missing information, mainly in the MSP.

In addition, 128 out of 152 patients had also retrievable samples collected at ICU admission (the acute phase).

The study protocol was approved by the Regional Ethical Review Board in Umeå and by the Swedish National Computer Data Inspection Board, and complies with the Declaration of Helsinki. All participants gave written informed consent for future use of data and blood samples.

### The septic event (acute phase)

Patients aged 18 years or older with a diagnosis of sepsis within 24 h after admittance to the intensive care unit (ICU) were included.

For patients with multiple admissions due to sepsis, only the first event was included. Sepsis, severe sepsis or septic shock was defined according to standard definitions [25]. Acute Physiology, Age and Chronic Health Evaluation (APACHE) II score was calculated and used for assessment of severity of illness at admission [26]. Sequential Organ Failure Assessment Score (SOFA) as a marker for organ dysfunction and disease severity was calculated at admission [27]. Data on height and weight were recorded, if present, and body mass index (BMI) was calculated as weight (kg) divided by height (m) squared. Data on length of stay, mortality, referral patterns, and reasons for admission, co-morbidities, and sources of infection, primary infection sites and causative microorganisms were collected. Microbiological cultures were considered relevant if acquired within 48 h before or after admission to the ICU. Pre-existing diseases were defined according to Knaus et al. [26].

### Clinical examinations at baseline (health survey)

In VIP and MONICA, participants were asked to complete a health questionnaire about their living conditions and cardiovascular risk factors, and anthropometry and blood pressure were measured. An oral glucose tolerance test with measurements of fasting and post-load glucose levels was routinely performed in VIP and in 60% of the MONICA participants but was not obtained in MSP and in NSMC. Diabetes and intermediate forms of glucose intolerance were determined according to WHO guidelines [28].

In the MONICA and MSP surveys, blood pressure was recorded in the sitting position after 5 min of rest, initially using a mercury sphygmomanometer but since 2004 by using semi-automatic devices (Omron M7, Omron Corp., Kyoto, Japan). In the VIP survey, blood pressure was measured after 5 min of rest in the recumbent position until 1 September 2009 and thereafter in the sitting position using devices as above. The measurements obtained in the recumbent position were adjusted according to a sex- and age-specific formula [29]. Hypertension was defined as systolic BP ≥140 mmHg and/or diastolic BP ≥90 mmHg and/or on anti-hypertensive medication.

Weight was measured without shoes in light indoor clothing and recorded to the nearest 0.2 kg. Height was measured to the nearest centimetre, without shoes, and BMI was calculated.

Subjects were categorized as daily smokers, ex-smokers or non-smokers. Total serum cholesterol was measured using a bench-top analyser (Reflotron^R^, Boehringer Mannheim GmbH Diagnostica, Mannheim, Germany) at the time of the health survey (VIP, until 1 September 2009) or by an enzymatic method (Boehringer Mannheim GmbH Diagnostica, Mannheim, Germany) at a central laboratory (MONICA and VIP after 1 September 2009). Cholesterol values obtained using the bench-top method were adjusted to the results measured at the central laboratory.

No clinical examinations were performed in the NSMC.

### Chemical analyses

Leptin and adiponectin were analysed in stored plasma obtained at the baseline health survey with a double-antibody radioimmunoassay method (Millipore Corporation, Billerica, MA, USA). The detectable level of the assays is 0.5 ng/mL. The total coefficient of variation (CV) for leptin was 4.7% at both low (2–4 ng/mL) and high (10–15 ng/mL) levels. For adiponectin, the total CV was 15.2% at low levels (2–4 μg/mL) and 8.8% at high (26–54 μg/mL) levels**.**

### Statistical analyses

Data are presented as numerical values or percentages. Non-normally distributed data were log_e_-transformed prior to analysis. Continuous data are presented as (geometric) means with 95% confidence intervals (CI). Pearson correlation or Spearman’s rho was used for test of associations. For comparisons, Fisher’s exact, Student t, or Mann–Whitney U-tests were used when appropriate. Paired sample t-test was used for intra-individual comparison. Since cases and referents had the same follow-up time within strata in this nested and matched case-referent study, logistic regression analysis (rather than Cox regression) using the conditional maximum likelihood routine designed for matched analysis was uses to estimate odds ratios (OR) and 95% CIs, and the influence of studied variables on future sepsis (stratified sex) was tested in univariable and multivariable models. Missing continuous values (BMI and serum cholesterol) were replaced with the cohort- and sex-specific median values representing the referents and missing values for categorical variables were treated as a separate category (not shown in tables). Non-conditional logistic regression analysis was used to calculate the risk for in-hospital death. Leptin and adiponectin were tested both as continuous (log_e_-transformed) and categorical (quartiles) variables. Cut-offs for quartiles were based on the cohort and sex-specific distribution of the baseline adipokine levels amongst referents (risk for sepsis) or amongst cases (risk for death). A *p*-value <0.05 was considered significant, and all *p*-values reported are two-sided. No adjustment was made for multiple testing. SPSS ver. 22 was used for statistical analysis.

## Results

### Subject characteristics

**Fig. 1:**
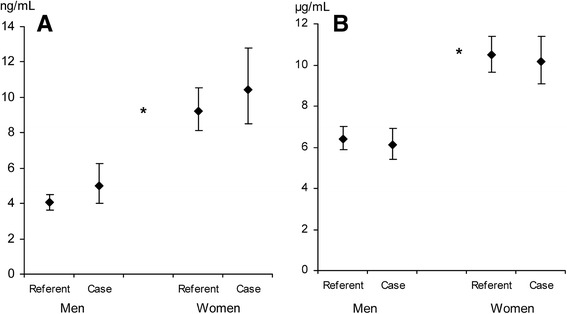
Leptin and adiponectin at baseline survey. Leptin (ng/mL, panel **a**) and adiponectin (μg/mL, panel **b**) levels at baseline survey for men and women, cases and referents (55/114 and 95/190, respectively). Data are presented as geometric means with 95% confidence intervals. * *p* < 0.001 between men and women

**Fig. 2:**
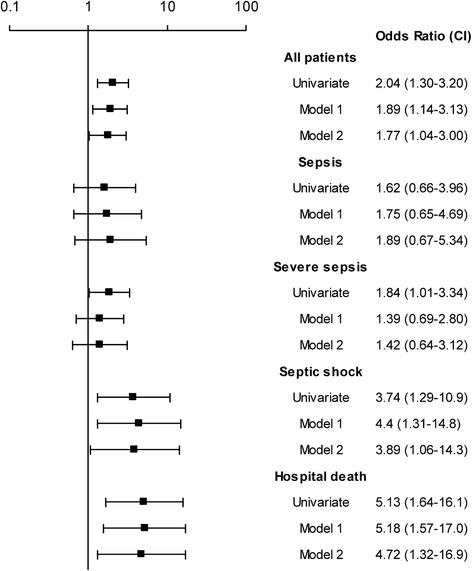
Leptin a baseline survey as predictor for sepsis. Values shown are odds ratios (OR) with 95% confidence intervals for leptin levels at baseline survey and the risk for a future sepsis event. Leptin were categorized into quartiles based on the cohort and sex-specific distribution amongst referents. The ORs represent the increased risk associated with the upper quartile of leptin (Q4) versus the reference (OR 1.00, lower three quartiles (Q1_3)). Model 1 included BMI, and model 2 included BMI, hypertension, reduced glucose tolerance (IFG, IGT a/o DM), and total cholesterol. Footnote: Cut-offs Q1_3 versus Q4 in absolute values for the different cohorts stratified for sex are for leptin (ng/mL): MONICA; 6.7 (only men): VIP; men 5.3, women 19.4: MSP 22.2 and NSMC 6.0 (only women in both) and for adiponectin (mg/mL): MONICA; 14.2 (only men): VIP; men 9.3, women 10.8: MSP 13.3 and NSMC 23.5 (only women in both)Abbreviations: MONICA, the Northern Sweden MONItoring Of trends and Determinants in CArdivascular Disease; VIP, Västerbotten Intervention Program; MSP, the Mammary Screening Program; NSMC, Northern Sweden Maternity Cohort

Subject with future sepsis had marginally higher BMI and lower total cholesterol than matched referents (Table 1). Prevalences of diabetes and hypertension, fasting and post-load blood glucose levels did not differ between cases and their referents. Circulating levels of leptin and adiponectin did not differ between cases and their referents. As expected, women had higher leptin (*P* < 0.001) and adiponectin (*P* < 0.001) levels (Figs. 1 and 2, respectively).

**Table 1:** Subject characteristics at baseline survey

	n = cases/referents	Cases	95% CI	Referents	95% CI	*p*
Age years	152/304	50.9	48.8–53.0	50.9	49.4–52.4	Matched
Female gender, %	152/304	62.5	54.6–69.8	62.5	56.9–67.8	Matched
BMI, kg/m^2^	118/233	27.1	26.2–28.1	26.1	25.6–26.6	0.05
Fasting glucose, mmol/L	82/157	5.5	5.2–5.8	5.4	5.3–5.6	0.41
Postload glucose, mmol/L	74/148	6.9	6.4–7.4	6.4	6.1–6.7	0.09
Reduced glucose tolerance, %	81/153	35.8	25.1–46.5	24.2	17.3–31.0	0.07
Daily smoker, % #	116/232	33.6	24.9–42.4	31.5	25.4–37.5	Matched
Systolic BP, mmHg	84/160	135	130–140	132	129–135	0.28
Diastolic BP, mmHg	84/160	82	80–85	82	80–83	0.60
Hypertension, %	84/160	56.0	45.1–66.8	43.1	35.4–50.9	0.06
Cholesterol, mmol/L¤	84/157	5.6	5.4–5.8	5.9	5.7–6.0	0.05
HDL cholesterol, mmol/L¤	26/59	1.17	0.89–1.54	1.1	1.00–1.2	0.60
Triglycerides, mmol/L¤	60/135	1.4	1.2–1.6	1.4	1.3–1.4	0.52

Values reported are means (¤geometric means) or percentages (%) with 95% CI. Hypertension was defined as systolic BP > 140 mmHg and/or diastolic BP > 90 mmHg and/or antihypertensive treatment. Reduced glucose tolerance included any of IFG, OGT or DM

*Abbreviations*: *DM* diabetes mellitus, *IFG* impaired fasting glucose, *IGT* impaired glucose tolerance (see text for definitions of DM, IFG and IGT), *HDL* high density lipoprotein, *BP* blood pressure

The acute event was classified as sepsis in 26%, as severe sepsis in 52% and as septic shock in 22%, with no differences between men and women (Table 2). The ICU mortality was 18% and the hospital mortality was 21%. The median time between survey participation and event was 6.5 years (IQR 7.7). The mean age at sepsis onset was 56.1 years in women and 61.0. years in men.The frequency of co-morbidities, including diabetes, steroid treatment, and renal insufficiency at the time of the sepsis event was similar in men and women (Table 2).

**Table 2:** Patient characteristics at ICU admission

	Men	Women	*p*
	*n* = 57	*n* = 95	
Age years	60.5(57.6–63.4)	56.3 (53.4–59.2)	0.02
Time between survey and sepsis years (median)	7.8 (IQR 8.3)	6.6 (IQR 8.1)	0.16
Disease scores			
APACHE II Score	18.2 (16.3–20.1)	18.9 (17.4–20.3)	0.57
SOFA score	7.0 (6.0–8.0)	7.0 (6.2–7.8)	0.95
Disease severity n (%)			
Sepsis	17 (29.8)	22 (23.2)	0.44
Severe sepsis	32 (56.1)	47 (49.5)	0.50
Septic shock	8 (14.0)	26 (27.4)	0.07
Hospital mortality	13 (22.8)	19 (20.0)	0.69
Co-morbidities n (%)			
COPD	1 (1.8)	3 (3.2)	1.00
Congestive heart failure	3 (5.3)	2 (2.1)	0.36
Chronic renal insufficiency	2 (3.5)	2 (2.1)	0.63
Diabetes n (%)			
Insulin treatment	4 (7.0)	7 (7.4)	1.00
Other treatments	4 (7.0)	5 (5.3)	0.73
Cancer n (%)			
Hematological	4 (7.0)	5 (5.3)	0.73
Localized	9 (15.8)	11 (11.6)	0.47
Metastatic	5 (8.8)	6 (6.3)	0.75
Immunosuppressants n (%)			
Chronic steroids	3 (5.3)	5 (5.3)	1.00
Chemotherapy	7 (12.3)	6 (6.3)	0.24
Other immunosuppression	4 (7.0)	7 (7.4)	1.00
Primary infection site n (%)			
Pneumonia	11 (19)	13 (14)	0.37
Abdominopelvic	19 (33)	31 (33)	1.00
Urinary tract	10 (18)	11 (12)	0.34
Other	17 (30)	38 (40)	0.23
Unknown	2 (3.5)	3 (3.2)	1.00

Values shown are means (95% CI), medians (IQR) and numbers (%)

*Abbreviations*: *APACHE* acute physiology, age and chronic health evaluation, *SOFA* sequential organ failure assessment, *CI* confidence interval, *IQR* interquartile range, *COPD* chronic obstructive pulmonary disease, *ICU* intensive care unit

### Correlations

At baseline, BMI correlated with high circulating levels of leptin (*r* = 0.36, *P* < 0.001), and with low circulating levels of adiponectin (*r* = −0.24, *P* < 0.001). In contrast, BMI did not associate with leptin (*r* = −0.05, *P* = 0.60) or with adiponectin (r = −0.05, *P* = 0.44) in the acute phase. Furthermore, leptin and adiponectin levels in the acute phase did not correlate with severity of disease expressed as APACHE- (leptin; *P* = 0.95, adiponectin; *P* = 0.79) or SOFA- scores (leptin; *P* = 0.88, adiponectin; *P* = 0.94). Leptin and adiponectin did not correlate at baseline (*P* = 0.14) or in the acute phase (*P* = 0.92).

### Predictors of sepsis at baseline

High circulating levels of leptin predicted a first-ever sepsis event (OR 2.04, 95% CI 1.30–3.2, *P =* 0.002) and retained predictive value after adjustment for BMI (OR 1.89, 95% CI 1.14–3.13, *P* = 0.01) and in the fully adjusted model (OR 1.77, 95% CI 1.04–3.00, *P* = 0.03) (Fig. 2). None of the included confounders remained significantly associated with sepsis in the fully adjusted model (data not shown). The septic event was graded as sepsis, severe sepsis or septic shock, and a graded response was seen in that there were stronger BMI-independent associations between hyperleptinemia and more severe forms of sepsis compared to milder forms of sepsis. Furthermore, leptin predicted sepsis-related in-hospital deaths (Fig. 2). Stratified for sex hyperleptinemia predicted sepsis in both men (OR 2.39, 95% CI 1.18–4.86, *P =* 0.02) and women (OR 1*.*83, 95% CI 1.02–3.27, *P =* 0.04). This association remained in men after adjustment for BMI (OR 2.60, 95% CI 1.20–5.61*, P =* 0.02), but not in women (*P* = 0.36). Furthermore, leptin predicted independently in-hospital death in men (OR 6.41 95% (1.32–31.1, *P =* 0.02). Adiponectin levels were not associated with the development of sepsis in any model (data not shown).

### Changes in leptin and adiponectin levels between baseline and the acute phase

**Fig. 3:**
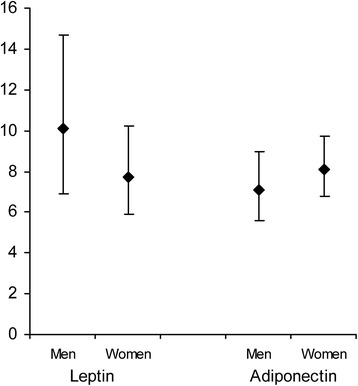
Leptin- and adiponectin in the acute phase. Leptin (ng/mL) and adiponectin (μg/mL) levels at the acute phase for men (*n* = 48) and women (*n* = 80). Data are presented as geometric means with 95% confidence intervals

**Fig. 4:**
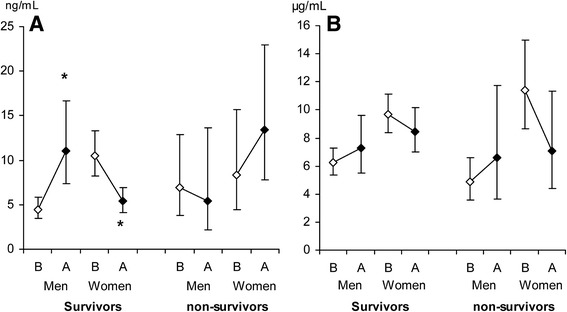
Leptin and adiponectin levels at baseline survey and in the acute phase stratified for hospital survival in men and women. Leptin (ng/mL, panel **a**) and adiponectin (μg/mL, panel **b**) levels at baseline survey (open diamond) and in the acute phase (filled diamond) for survivors (men *n* = 37, women *n* = 62) and non-survivors (men *n* = 11, women *n* = 18). Data are presented as geometric means with 95% confidence intervals. B = baseline survey; A = acute phase. * p < 0.001 between baseline and acute phase

Contrary to baseline, there were no differences in leptin- or adiponectin levels between men and women in the acute phase (leptin; *P* = 0.26, adiponectin; *P* = 0.36), (Fig. 3). However, the increase in both leptin- and adiponectin levels between baseline and the acute phase were significantly higher in men than in women (leptin *P* = 0.001, adiponectin *P* = 0.02). Leptin levels increased from baseline to the acute phase in surviving men (*P* = 0.001) whereas surviving women decreased their leptin levels from baseline to the acute phase (*P* = 0.02) (Fig. 4a). There was no significant change in leptin levels from baseline to the acute phase in non-survivors (men; *P* = 0.09, women; *P* = 0.31) (Fig. 4a).

Adiponectin levels increased from baseline to the acute phase in both surviving and non-surviving men whereas surviving and non-surviving women decreased their adiponectin levels from baseline to the acute phase, however the changes were not statistically significant (Fig. 4b).

### Predictors of in-hospital death

**Fig. 5:**
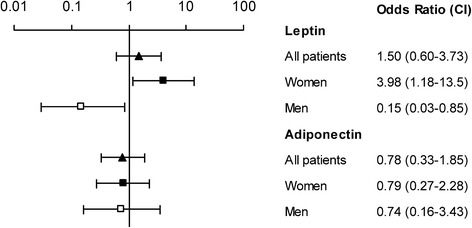
Leptin and adiponectin in acute phase as predictors for in-hospital death. Values shown are Odds ratios (OR) with 95% confidence intervals for leptin and adiponectin concentrations in the acute phase and risk for in-hospital death for all (filled triangle, *n* = 128), for women (filled square, *n* = 80), and for men (open square, n = 48). Leptin and adiponectin were categorized into quartiles based on the cohort and sex-specific distribution amongst cases at baseline. The ORs represent the increased risk associated with the higher three quartiles (Q2_4) versus the reference (OR 1.00, the lowest Q1). Footnote: Cut-offs Q1 versus Q2_4 in absolute values for the different cohorts stratified for sex are for leptin (ng/mL): MONICA; 5.7 (only men): VIP; men 2.6, women 6.5: MSP 8.0 and NSMC 1.7 (only women in both) and for adiponectin (mg/mL): MONICA; 5.2 (only men): VIP; men 4.1, women 6.2: MSP 7.4 and NSMC 6.8 (only women in both). Abbreviations: MONICA, the Northern Sweden MONItoring Of trends and Determinants in CArdivascular Disease; VIP, Västerbotten Intervention Program; MSP, the Mammary Screening Program; NSMC, Northern Sweden Maternity Cohort

High leptin levels expressed as the highest three quartiles compared to the lowest quartile predicted death irrespective of disease severity (APACHE II or SOFA score) in women (OR 4.18, 95% CI 1.17–15.00, *P* = 0.03), whereas high leptin levels in men were associated with a decreased risk of death (OR 0.05, 95% CI 0.01–0.48, *P =* 0.01) (Fig. 5). Adjustment with SOFA score gave similar point estimates. Furthermore, leptin levels were dichotomised using a single cut-off for both men and women (10 ng/mL). Levels above 10 ng/mL were significantly associated with in hospital death, OR 2.48, 95% (1.06–5.77, *P* = 0.035), which remained after adjustment for APACHE II; OR 2.68, 95% CI (1.08–6.65), *P* = 0.033, but not after adjustment for SOFA score, OR 2.15 95% CI (0.86–5.39), *P* = 0.10.

This risk for in-hospital death remained in women, OR 7.48, 95% CI (2.30–24.28), *P* = 0.001, but not in men, OR 0.49, 95% CI (0.12–1.95), *P* = 0.31. When analyzed as a continuous log_e_-transformed variable, high leptin in the acute phase predicted in-hospital death independently of APACHE II-score in women (OR 1.58, 95% CI 1.01–2.48, *P* = 0.04), whereas the protective effect of high leptin levels in men did not remain significant (OR 0.80, 95% CI 0.42–1.49, *P =* 0.48).

APACHE II and SOFA scores remained associated with in-hospital death after adjustment for leptin levels in the acute phase (*P* < 0.001 for both APACHE and SOFA).

Adiponectin did not associate with in-hospital death, neither as a categorical variable, nor as a continuous variable (Fig. 5).

## Discussion

To our knowledge, this is the first study to prospectively analyse serum levels of leptin and adiponectin in patients who subsequently develop sepsis. Furthermore, we are not aware of any previous studies of intra-individual changes in adipokine levels from a non-septic basal state to an acute septic state and the associations with outcome with a possible sex-difference.

Here we report that high levels of circulating leptin at baseline independently predicted a first-ever sepsis event, possibly with a sex-related difference. Furthermore, there was a graded response, with stronger associations between hyperleptinemia at baseline and more severe forms of sepsis compared to milder forms of sepsis. In contrast to baseline, levels of adipokines did not differ between men and women in the acute phase, primarily due to a higher increase in adipokine levels in men than in women. Furthermore, increased leptin levels in the acute phase were associated with better survival in men but not in women.

In contrast, adiponectin at baseline was not associated with the development of sepsis and adiponectin levels in the acute phase were not associated with outcome.

Our results are consistent with earlier reports showing that high leptin levels are related to better outcome in acute sepsis [15, 16, 18, 30]. However, we saw this association in men.

In contrast, we found that high leptin levels at baseline predicted sepsis with an unfavourable outcome. This apparent contradiction might reflect the pleiotropic properties of adipokines. Leptin and adiponectin are adipocyte-derived cytokines with pro- and anti-inflammatory effects, linking adipose tissue with metabolism and inflammation [31, 32]. Baseline leptin and adiponectin levels reflect the metabolic conditions at the time and are determined by nutritional status and correlate to the amount of fat mass, where leptin increase and adiponectin decrease with increasing obesity. These aberrations relate to the development of cardiovascular disease (CVD) and diabetes [33, 34], possibly by inducing endothelial dysfunction [35].

Hyperleptinemia also induces a low-grade pro-inflammatory state where cells in the innate immune system are activated and release cytokines. Furthermore, activation of the innate immune system induces leptin secretion. Thus, there are bidirectional loops between the inflammatory response and leptin. A key-feature in the sepsis syndrome is the acute systemic inflammation with capillary leakage and vasoplegia, which leads to severe hypotension and septic shock. It is reasonable to believe that patients with obesity-induced low grade inflammatory state with impaired endothelial function, may have an unfavourable outcome from a superimposed infection with accompanying endothelial activation and the consequent development of severe sepsis or septic shock [35–37]. However, in the acute phase, the ability to secret sufficient amounts of adipokines may be beneficial by activating the immune response.

In the acute phase, the expected association between BMI and leptin was not seen, and the difference in circulating levels between men and women was attenuated, indicating that leptin is not only a simple measure of obesity but also a complex hormone with acute-phase properties [32, 38]. The equalization of adipokine levels between sexes in the acute phase was mainly due to a greater increase in men. Equalization in leptin levels between sexes have also been reported in children with sepsis and that children with higher increases in leptin levels had an unfavourable outcome [39]. An additional increase in leptin levels from initially high levels at baseline may not be beneficial from a physiologic perspective or may indicate leptin resistance [34, 40].The differences in changes in adipokine levels in the acute septic state between men and women may in part explain conflicting results from previous studies.

We found sex-differences in the predictive value of leptin with stronger association in men. The design of this study does not allow an explanation for these gender differences, but may be due to sex-differences in leptin signalling [41, 42] and we have reported similar sex-related differences in leptins ability to predict stroke and diabetes [43, 44].

This study has limitations. The cases were not entirely representative of all consecutive patients admitted to the ICU as participation in a health survey prior to the sepsis event was mandatory. Two of the health surveys, the MSP and the NSMC, included only women. Further, the mean age in the NSMC was low, which explains why most our cases were (younger) women. This contrasts with most sepsis studies with a majority of men [45]. However, we believe that the matching procedure with the cases and their two referents with the same sex and within the same cohort, as well as being able to compare inter individual adipokine levels in health (baseline survey) with levels in the acute septic condition (acute phase) is a strength to this study.

The protocol for these health surveys did not include analysis of CRP, HbA1c, C-peptide or insulin and it has not been done in the present study due to limited sample volumes.

We did not exclude patients with diabetes, renal failure, patients with steroid treatment, or those with other immunosuppressive conditions; conditions that may affect adipokine levels. We thus believe that our selection represents the clinical reality. Further, the frequency of co-morbidities that could affect adipokine levels did not differ between men and women.

Although there is a strong correlation between circulating levels of adipokines and BMI, there is a significant inter-individual variation. As our prediction analysis is adjusted for BMI, the results indicate that leptin has effects independent from just being a measure of the amount of adipose tissue, an effect more pronounced in men. Despite shortcomings as a measure of fat mass, BMI still used because of its simplicity and reproducibility. The rationale for matching on smoking status was the higher risk of perioperative infectious complications related to smoking, and that smoking influence leptin levels [46, 47]. This matching was not complete due to missing information, especially in the NSMC cohort. Repeated analysis after omitting the NSMC- or MSP cohort did not alter the results and leptin remained predictive of an event of sepsis.

Due to reduced sample size in the acute sepsis event (*n* = 128) the chosen cohort- and sex specific cut of levels for adipokines (Q1 vs Q2–4) differ from the cut of levels in the prediction analysis (Q4 vs Q1–3) (baseline samples *n* = 152). Using cut offs of 10 ng/mL for leptin as used by others gave similar results as using cohort-and sex specific Q1 vs Q2–4, with risk for in-hospital death in women and protection in men (although not significant). However, 10 ng/ml is a high leptin level in men and most patients will end up in the comparator group using this approach. As leptin physiology probably differs between men and women we argue that sex-specific cut-offs are important and the strengths of our study is the sample size which allows us to stratify the analysis for sex.

Circulating levels of leptin and adiponectin show diurnal variation and sampling at the baseline survey did not take this into account. However, if any effect this would diminish the associations found in this study. Furthermore, the samples from the pre-sepsis investigation do represent population-based levels and we have previously shown that individual leptin levels are stable over long periods of time in men and women and in different ethnicities [48]. Leptin is also stable after long-time storage of samples [49].

## Conclusion

We conclude that hyperleptinemia at baseline predicts a first-ever sepsis event, even after adjustment for BMI and other known cardiovascular risk factors. There is a graded response between high levels of leptin at baseline and sepsis severity. In contrast, hyperleptinemia in the acute phase relates to a reduced risk for in-hospital death in men whereas hyperleptinemia relates to an increased risk in women. Furthermore, we found that inertia in the leptin response to sepsis in the acute phase was related to worse outcome, which is an intriguing finding that could be of outermost importance for the understanding of the triggers of an adequate immune response to a life-threatening sepsis.
